# Supplementary figures and images for: Dynamic assembly of malate dehydrogenase–citrate synthase multienzyme complex in the mitochondria
Source: eLife. 2026 Jul 8;14:RP107953. doi: 10.7554/eLife.107953 (PMC13345634; doi:10.7554/eLife.107953)

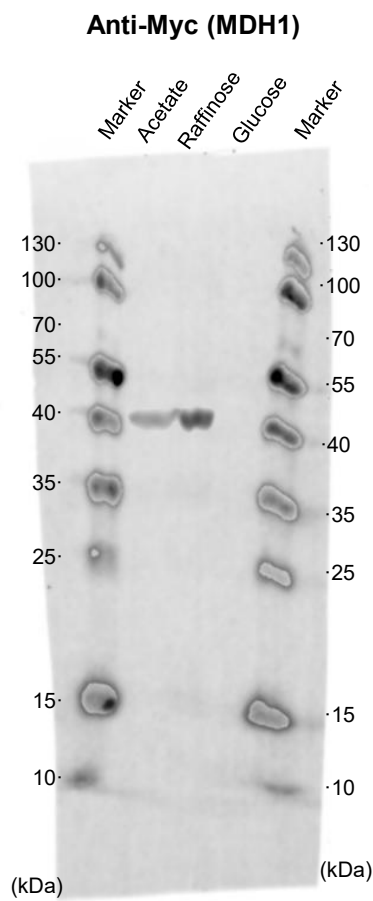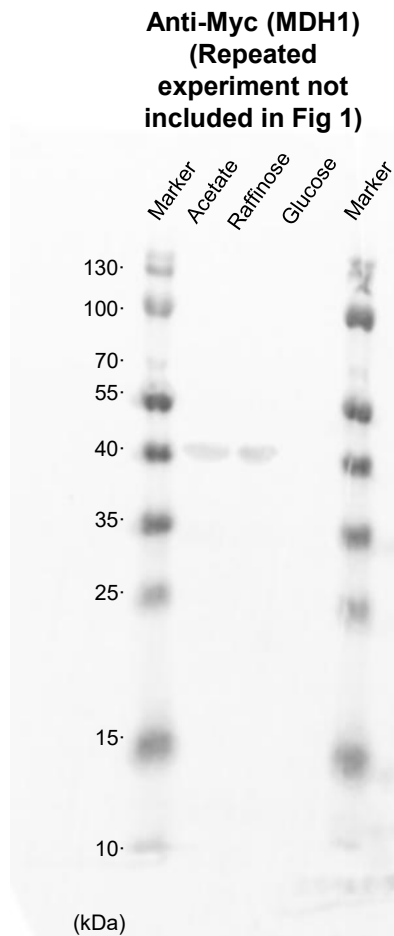

**Anti-HA (CIT1)**

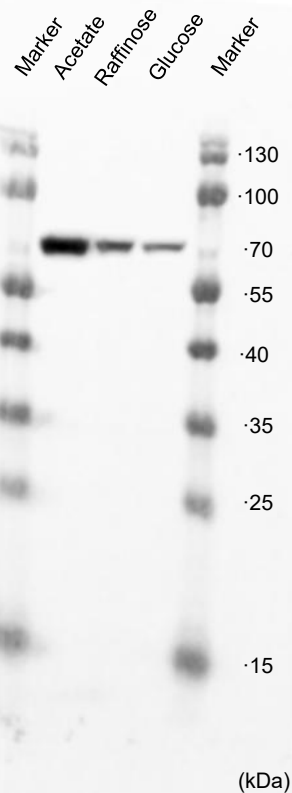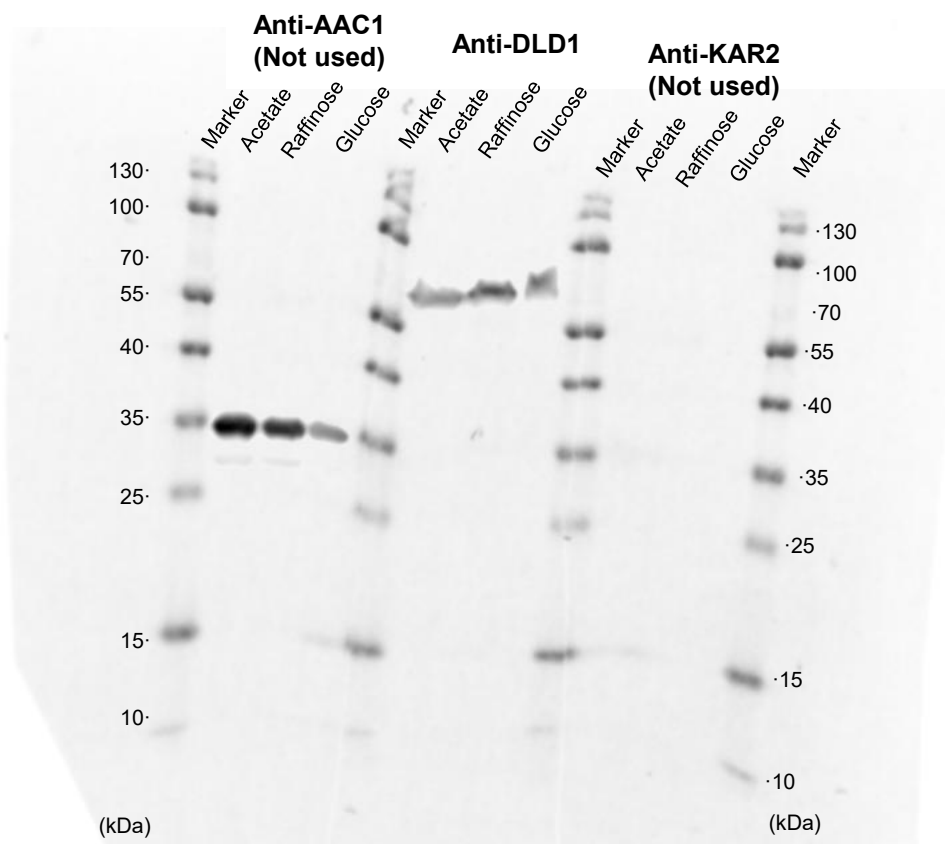

Supplement: Figure 1—source data 1. [file elife-107953-fig1-data1.pdf]

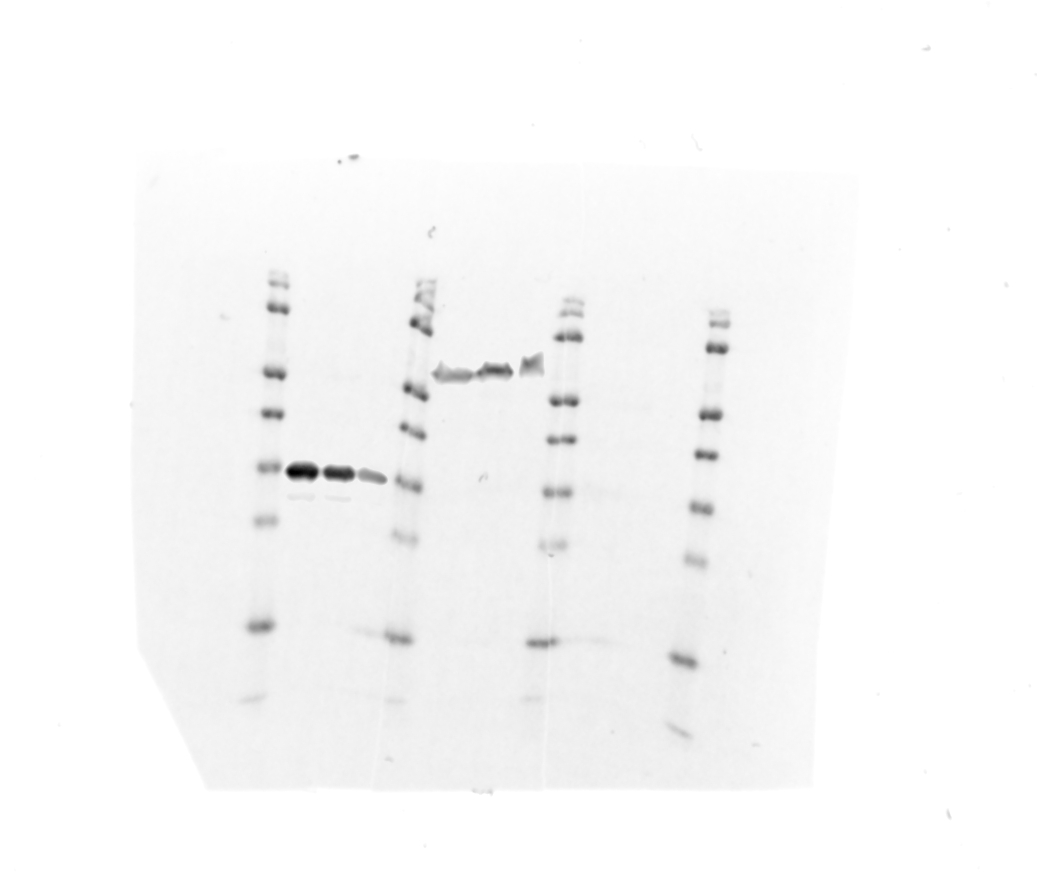

Supplement: Figure 1—source data 2. [file elife-107953-fig1-data2.zip › Aac2_ Drd1 and Kar2 Loading Ctrls 03202026.jpg]

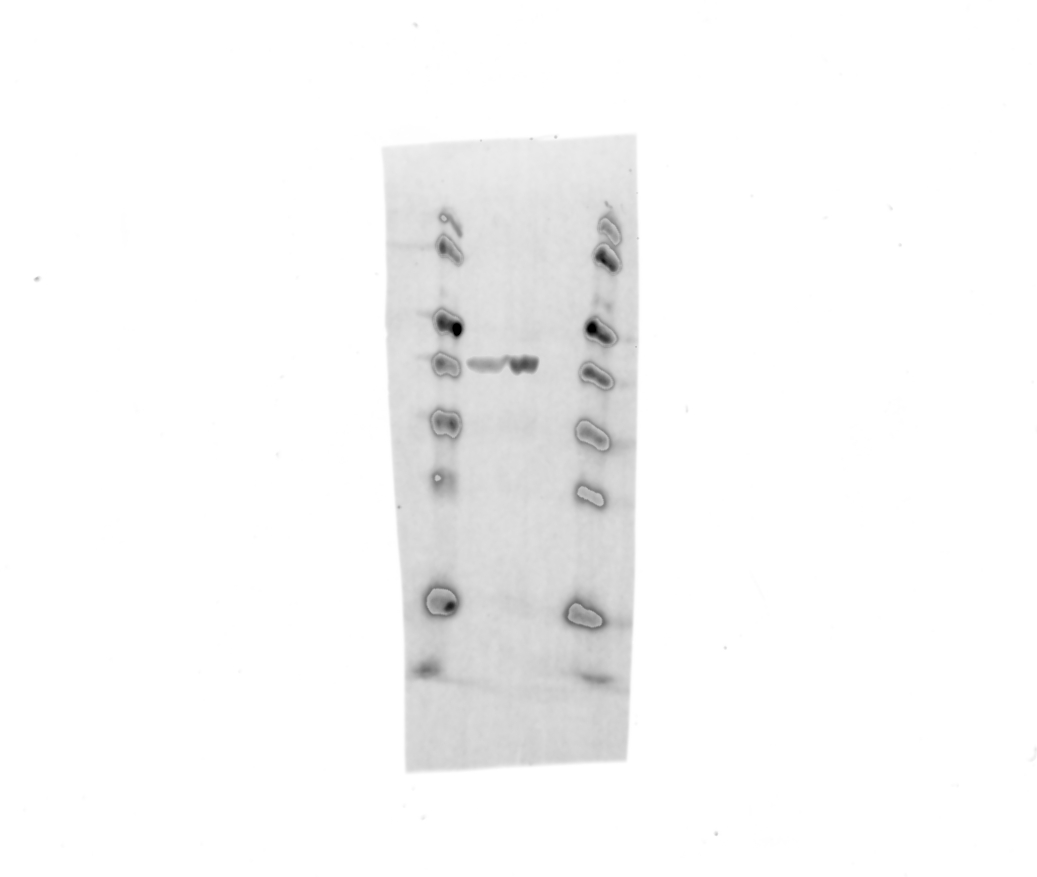

Supplement: Figure 1—source data 2. [file elife-107953-fig1-data2.zip › Mdh1 expression - different carbon sources 03012026.jpg]

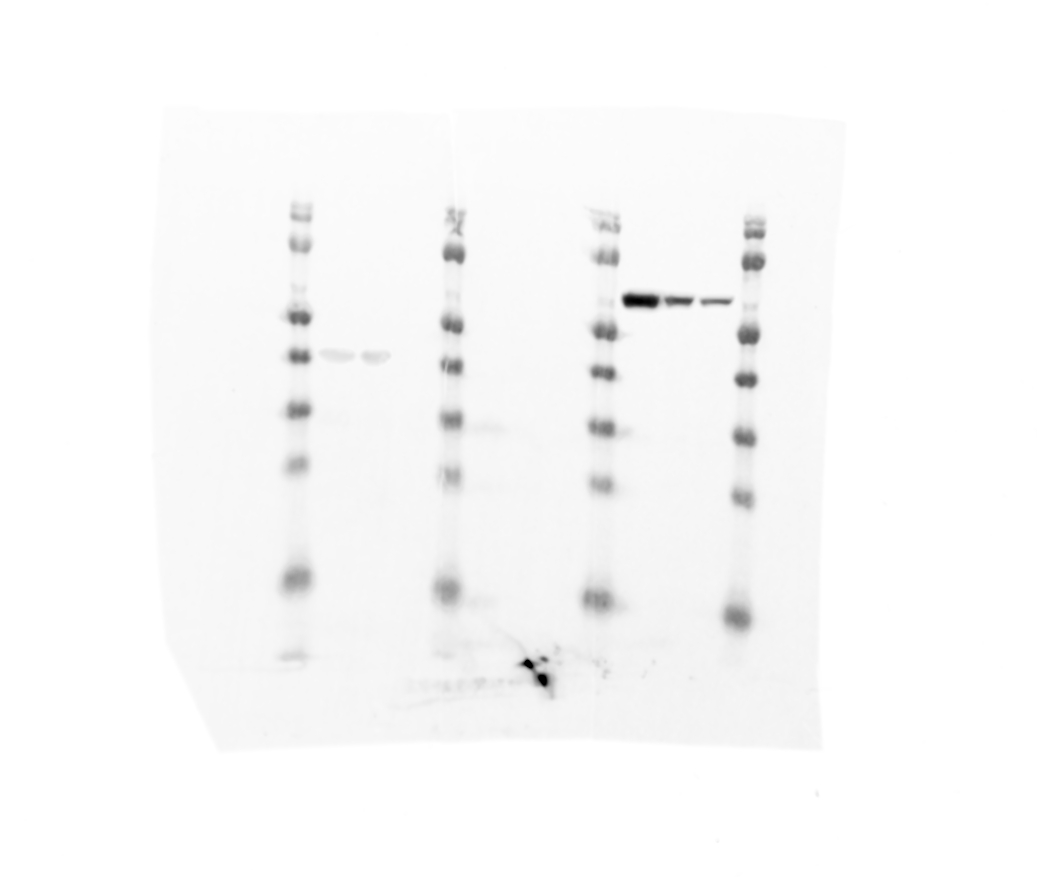

Supplement: Figure 1—source data 2. [file elife-107953-fig1-data2.zip › Mdh1-Cit1 expression - different carbon sources 02262026.jpg]

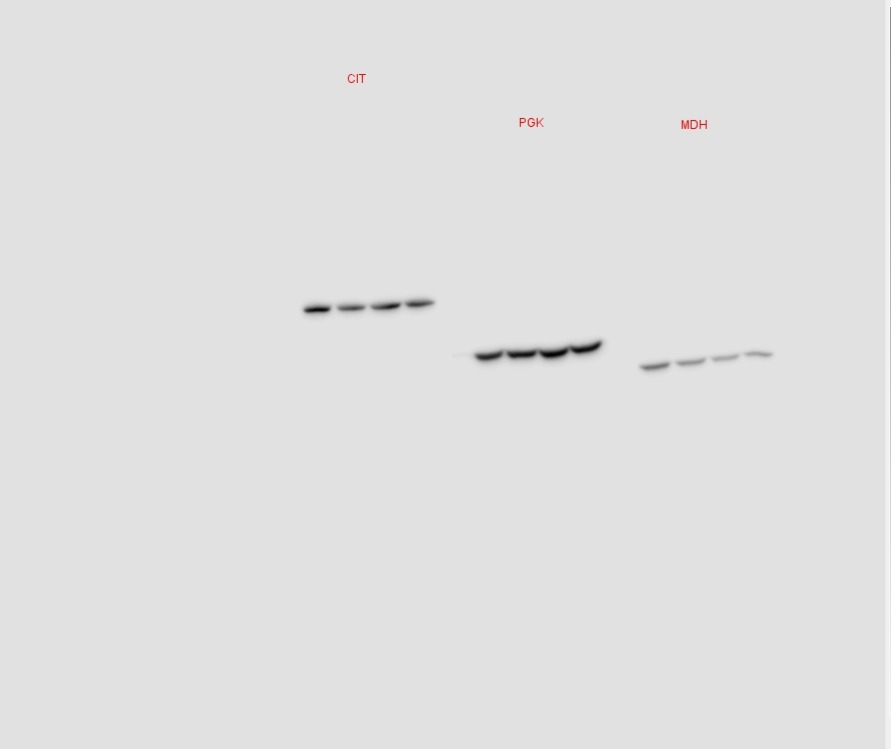

Supplement: Figure 2—figure supplement 1—source data 2. [file elife-107953-fig2-figsupp1-data2.zip › Fig2sup1C.JPG]

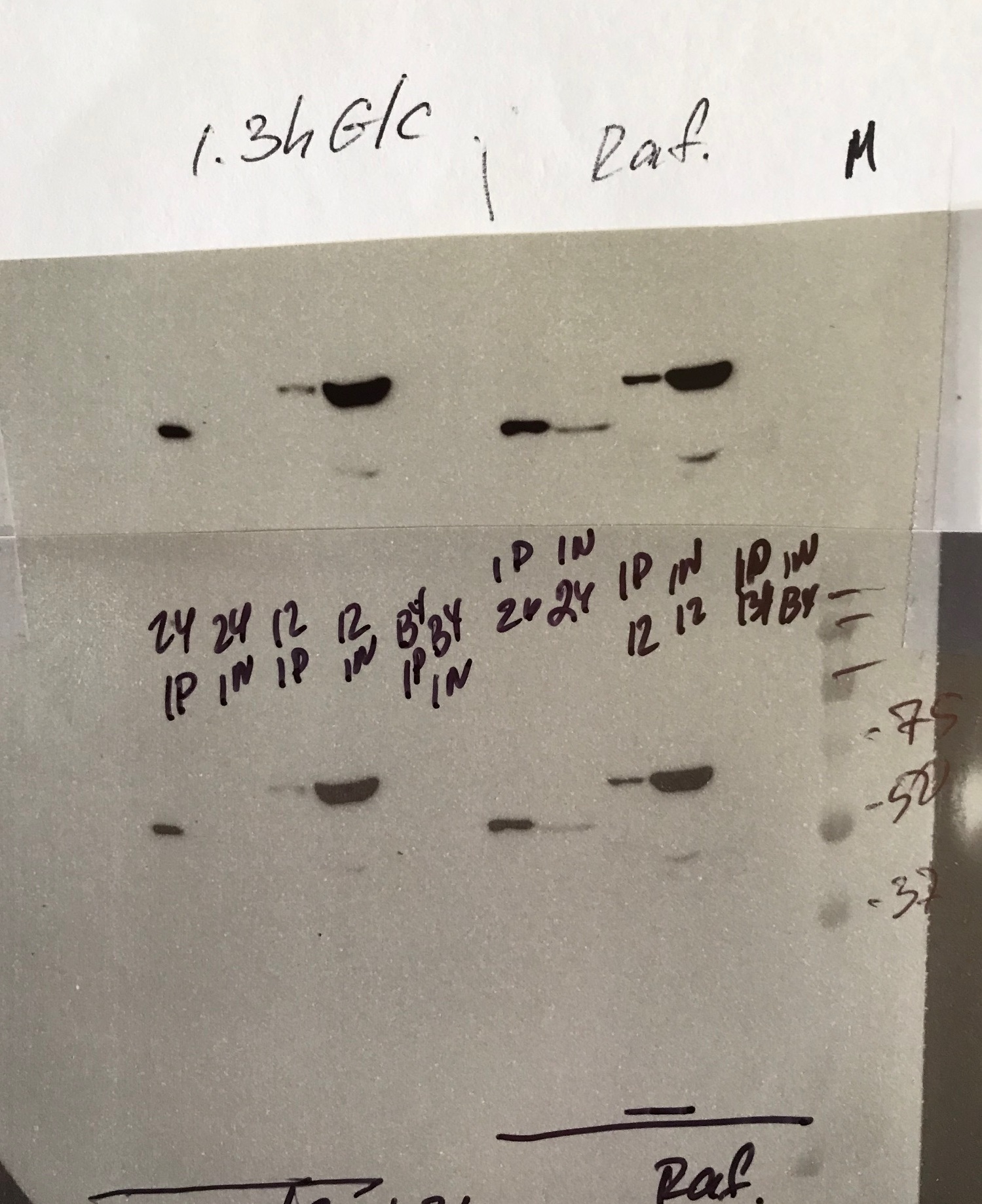

Supplement: Figure 2—figure supplement 1—source data 2. [file elife-107953-fig2-figsupp1-data2.zip › Fig2sup1A1.jpg]

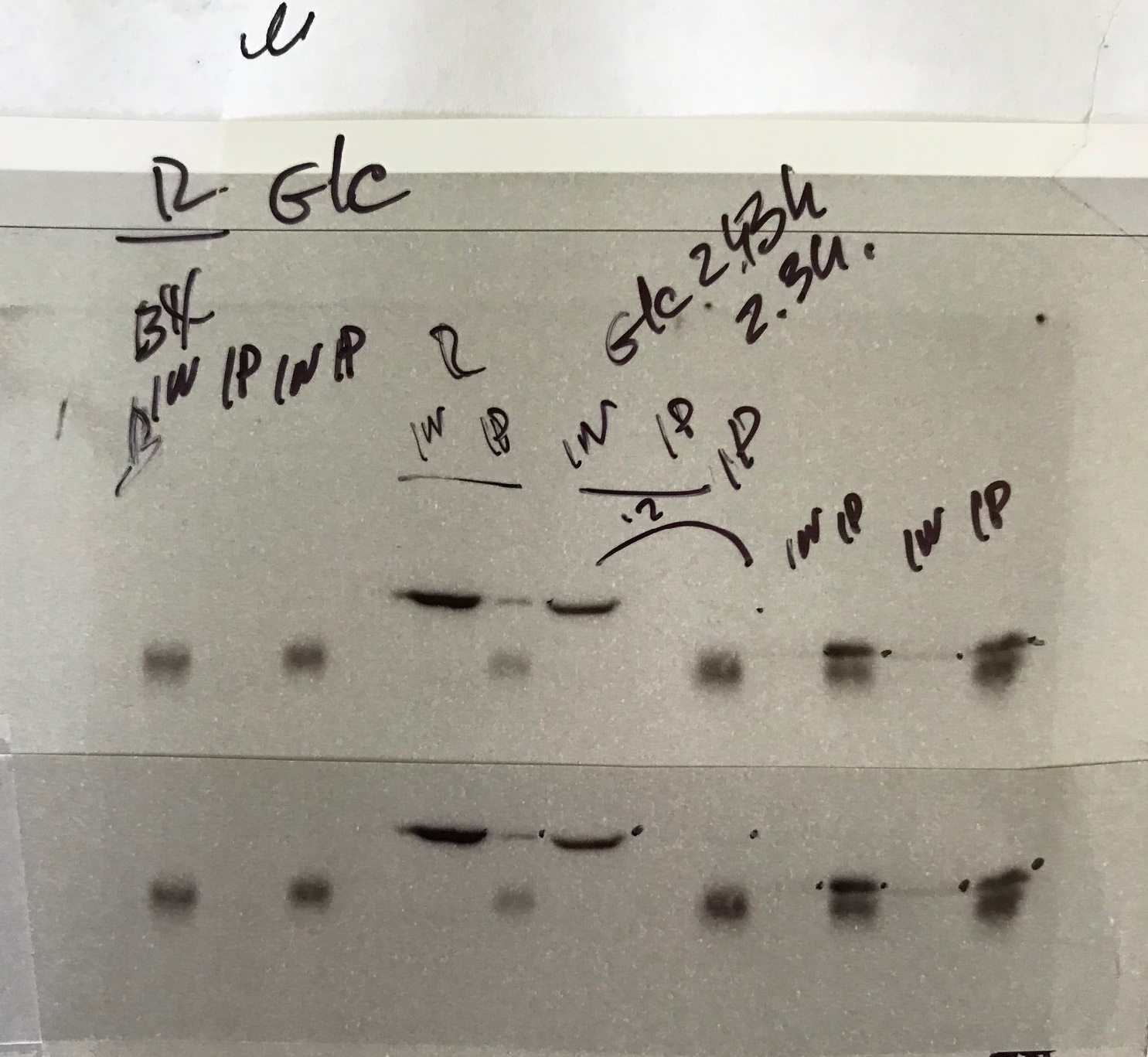

Supplement: Figure 2—figure supplement 1—source data 2. [file elife-107953-fig2-figsupp1-data2.zip › Fig2sup1A2.jpg]
