# Supplementary material for: Dynamic assembly of malate dehydrogenase–citrate synthase multienzyme complex in the mitochondria
Source: eLife. 2026 Jul 8;14:RP107953. doi: 10.7554/eLife.107953 (PMC13345634; doi:10.7554/eLife.107953)

Figure 2 – figure supplement 1 A

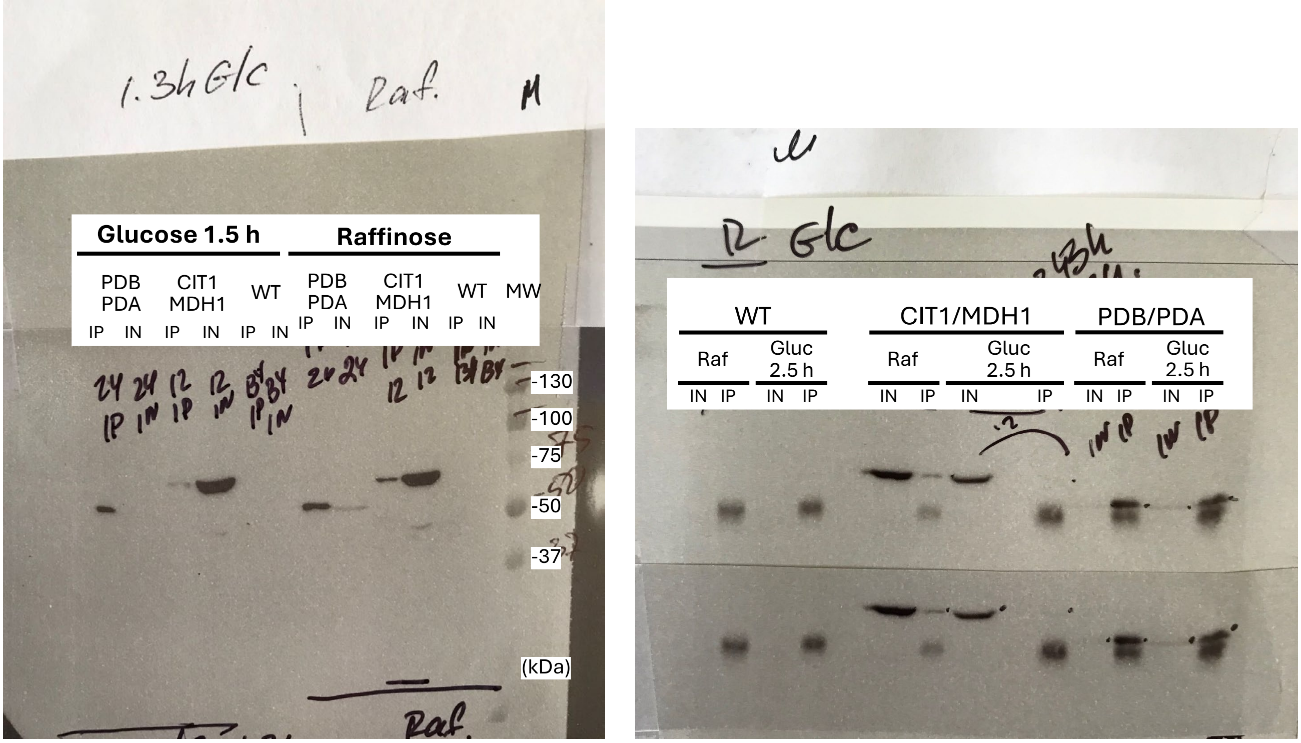

Figure 2 – figure supplement 1 C

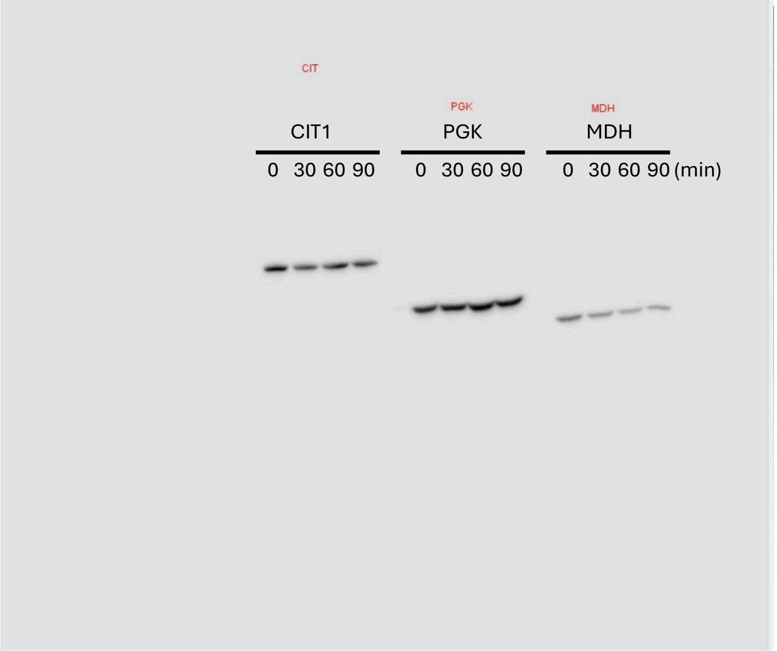

Supplement: Figure 2—figure supplement 1—source data 1. [file elife-107953-fig2-figsupp1-data1.pdf]
