# Supplementary material for: Dynamic assembly of malate dehydrogenase–citrate synthase multienzyme complex in the mitochondria
Source: eLife. 2026 Jul 8;14:RP107953. doi: 10.7554/eLife.107953 (PMC13345634; doi:10.7554/eLife.107953)
Supplement: Peer review history

# Editor report: eLife Assessment

**DOI:** 10.7554/eLife.107953.3.sa0

**Authors:** P Darrell Neufer

**Affiliations:** Wake Forest University School of Medicine United States

**Keywords:** Compelling, Important

This **important** study provides novel information on multi-enzyme complexes, known as metabolons, that form between sequential enzymes in a metabolic pathway. Using an innovative NanoBiT split-luciferase system, the authors present **compelling** evidence that malate dehydrogenase (MDH1) and citrate synthase (CIT1) dynamically associate under different metabolic conditions in *Saccharomyces cerevisiae*. The findings suggest the dynamic MDH1-CIT1 interaction facilitates control of TCA pathway flux rate.

# Referee report: Reviewer #1 (Public review)

**DOI:** 10.7554/eLife.107953.3.sa1

Summary:

The study by the Obata group characterizes the dynamics of the canonical malate dehydrogenase-citrate synthase metabolon in yeast.

Strengths:

The study is well-written and appears to give clear demonstrations of this phenomenon.

Studies of the dynamics of metabolon formation are rare; if the authors can address the concern detailed below, then they have provided such for one of the canonical metabolons in nature.

Weaknesses:

There is a fundamental issue with the study, which is that the authors do not provide enough support or information concerning the split luciferase system that they use. Is the binding reversible or not? How the data is interpreted is massively influenced by this fact. What are the pros and cons of this method in comparison to, for example, FLIM-FRET? The authors state that the method is semi-quantitative - can they document this? All of the conclusions are based on the quality of this method. I know that it has been used by others, but at least some preliminary documentation to address these questions is required.

Comments on revised version:

I feel that the authors have adequately addressed my prior concerns. I have no further critiques of their work.

# Referee report: Reviewer #2 (Public review)

**DOI:** 10.7554/eLife.107953.3.sa2

This study explores the dynamic association between malate dehydrogenase (MDH1) and citrate synthase (CIT1) in *Saccharomyces cerevisiae*, with the aim of linking this interaction to respiratory metabolism. Utilizing a NanoBiT split-luciferase system, the authors monitor protein-protein interactions in vivo under various metabolic conditions.

Major Concerns:

(1) NanoBiT Signal May Reflect Protein Abundance Rather Than Interaction Strength

In Figure 1C, the authors report increased MDH1-CIT1 interaction under respiratory (acetate) conditions and decreased interaction during fermentation (glucose), as indicated by NanoBiT luminescence. However, this signal appears to correlate strongly with the expression levels of MDH1 and CIT1, raising the possibility that the observed luminescence reflects protein abundance rather than specific interaction dynamics. To resolve this, NanoBiT signals should be normalized to the expression levels of both proteins to distinguish between abundance-driven and interaction-driven changes.

(2) Lack of Causal Evidence

The study presents a series of metabolic perturbation experiments (e.g., arsenite, AOA, antimycin A, malonate) and correlates changes in metabolite levels with NanoBiT signals. However, these data are correlative and do not establish a functional role for the MDH1-CIT1 interaction in metabolic regulation. To demonstrate causality, the authors should implement approaches to specifically disrupt the MDH1-CIT1 interaction. One strategy could involve using a 15-residue peptide (Pept1) derived from the Pro354-Pro366 region of CIT1, previously shown to mediate the interaction or introducing the cit1Δ3 (Arg362Glu) mutation, which perturbs binding. Metabolic flux analysis using ^13C-labeled glucose and mitochondrial respiration assays (e.g., Seahorse) could then assess functional consequences.

(3) Absence of Protein Expression Controls Under Perturbation Conditions

In experiments involving acetate, arsenite, AOA, antimycin A, and malonate, the authors infer changes in MDH1-CIT1 association based solely on NanoBiT signals. However, no accompanying data are provided on MDH1 and CIT1 protein levels under these conditions. This omission weakens the conclusions, as altered expression rather than interaction strength could underlie the observed luminescence changes. Immunoblotting or quantitative proteomics should be used to confirm constant protein expression across conditions.

Conclusion:

Although the central question is compelling and the use of NanoBiT in live cells is a strength, the manuscript requires additional experimental rigor. Specifically, normalization of interaction signals, introduction of causative perturbations, and validation of protein expression are essential to substantiate the study's claims.

Comments on revised version:

The manuscript is much improved.

# Referee report: Reviewer #3 (Public review)

**DOI:** 10.7554/eLife.107953.3.sa3

Summary:

Metabolons are multisubunit complexes that promote the physical association of sequential enzymes within a metabolic pathway. Such complexes are proposed to increase metabolic flux and efficiency by channeling reaction intermediates between enzymes. The TCA cycle enzymes malate dehydrogenase (MDH1) and citrate synthase (CIT1) have been linked to metabolon formation, yet the conditions under which these enzymes interact, and whether such interactions are dynamic in response to metabolic cues, remains unclear, particularly in the native cellular context. This study uses a nanoBIT protein-protein interaction assay to map the dynamic behavior of the MDH1-CIT1 interaction in response to multiple metabolic stimuli and challenges in yeast. Beyond mapping these interactions in real time, the authors also performed GC-MS metabolomics to map whole cell metabolite alterations across experimental conditions. Finally, the authors use microscale thermophoresis to determine components that alter the MDH1-CIT1 interaction in vitro. Collectively, the authors synthesize their collected data into a model in which the MDH1-CIT1 metabolon dissociates in conditions of low respiratory flux, and is stimulated during conditions of high respiratory flux. While their data largely support these models, some key exceptions are found that suggest this model is likely oversimplified and will require further work to understand the complexities associated with MDH1-CIT1 interaction dynamics. Nonetheless, the authors put forth an interesting and timely toolkit to begin to understand the interaction kinetics and dynamics of key metabolic enzymes that should serve as a platform to begin disentangling these important yet understudied aspects of metabolic regulation.

Strengths:

- The authors address an important question: how do metabolon-associated protein protein interactions change across altered metabolic conditions?

- The development and validation of the MDH1-CIT1 nanoBIT assay provides an important tool to allow the quantification of this protein-protein interaction in vivo. Importantly, the authors demonstrate that the assay allows kinetic and real time assessment of these protein interactions, which reveal interesting and dynamic behavior across conditions.

- The use of classic biochemical techniques to confirm that pH and various metabolites can alter the MDH1-CIT1 interaction in vitro is rigorous and supports the model put forth by the authors.

Weaknesses:

The authors have addressed identified weaknesses within the revision of their manuscript.

# Author comment: Author response

**DOI:** 10.7554/eLife.107953.3.sa4

**Authors:** Joy Omini, Inga Krassovskaya, Taiwo Adeolu Dele-Osibanjo, Connor Pedersen, Toshihiro Obata

**Affiliations:** University of Nebraska-Lincoln Lincoln United States; University of Nebraska-Lincoln Lincoln United States; University of Nebraska-Lincoln Lincoln United States; University of Nebraska-Lincoln Lincoln United States; University of Nebraska-Lincoln Lincoln United States

The following is the authors’ response to the original reviews.

**eLife Assessment**

This study reports a dynamic association/dissociation between malate dehydrogenase (MDH1) and citrate synthase (CIT1) in *Saccharomyces cerevisiae* under different metabolic conditions that control TCA pathway flux rate. The research question is timely, the use of the NanoBiT split-luciferase system to monitor protein-protein interactions is innovative, and the significance of the findings is valuable. However, the strength of evidence needed to support the conclusions was found to be incomplete based on a lack of critical control and mechanistic experiments.

We thank the editor for this thoughtful assessment of our work. We are encouraged that the research question, experimental approach, and overall significance were viewed positively.

To address the concern regarding the strength of evidence, we have implemented additional controls in the revised manuscript. Specifically, we have repeated all MDH1CIT1 interaction measurements alongside strains expressing full-length NanoLUC fusion proteins to assess MDH1 and CIT1 protein abundance. The resulting data, now included as supplementary figures (Figure 2 – figure supplement 2, Figure 2 – figure supplement 3, Figure 3 – figure supplement 1, Figure 4 – figure supplement 2), demonstrate the reproducibility of the findings and indicate that the observed changes in MDH1-CIT1 interaction are not attributable to protein abundance variations.

We agree that a detailed mechanistic dissection of how the MDH1–CIT1 complex influences metabolic pathway flux is an essential piece of evidence for establishing the functions of the metabolon. However, such analyses require extensive additional investigation beyond the scope of the present study. Accordingly, we have clarified the aims of this work in the revised manuscript to emphasize that our primary objective is to characterize the dynamic behavior of the MDH1–CIT1 interaction under different metabolic conditions and to identify key factors associated with its regulation.

We believe these revisions strengthen the rigor of the study, better define its scope, and provide a solid foundation for future mechanistic investigations.

**Public Reviews:**

**Reviewer #1 (Public review):**

Summary:

The study by the Obata group characterizes the dynamics of the canonical malate dehydrogenase-citrate synthase metabolon in yeast.

Strengths:

The study is well-written and appears to give clear demonstrations of this phenomenon.

Studies of the dynamics of metabolon formation are rare; if the authors can address the concern detailed below, then they have provided such for one of the canonical metabolons in nature.

We sincerely thank the reviewer for their positive assessment and for recognizing the value of our study in characterizing the dynamics of the MDH1-CIT1 metabolon. We appreciate the recognition that studies of metabolon dynamics are rare and that our work provides a clear demonstration of this phenomenon for a canonical metabolon. We have carefully addressed the methodological concerns regarding the NanoBiT system as detailed below to further strengthen the evidence for our findings.

Weaknesses:

There is a fundamental issue with the study, which is that the authors do not provide enough support or information concerning the split luciferase system that they use.

We agree that a detailed description of the NanoBiT system is essential to ensure the reliability of the methodology. As suggested, we have added a dedicated paragraph to the Introduction (Lines 90–103) to clarify these technical aspects, supported by the foundational work of Dixon et al. (2016).

Is the binding reversible or not? How the data is interpreted is massively influenced by this fact.

Yes, the NanoBiT system is specifically designed to be reversible. The intrinsic affinity of the subunits is low (*K*_D_ = 190 μM), and the association and dissociation rate constants (*k*_on_ = 500 M^-1^s ^-1^, *k*_off_ = 0.2 s^-1^) are well outside the range of typical protein-protein interactions (Dixon et al., 2016). These kinetics ensure that the assembly and disassembly of the luminescent complex are dictated solely by the interaction characteristics of the target proteins (MDH1 and CIT1) and not by the tags themselves. This allows for real-time monitoring of both the association and dissociation phases.

What are the pros and cons of this method in comparison to, for example, FLIM-FRET?

We have now explicitly addressed the pros and cons of our methodology compared to fluorescence-based systems:

Pros: The NanoLUC-based reporter is 150 times brighter than conventional luciferases and has a significantly higher dynamic range (Hall et al 2016), allowing detection of weak transient interactions. Importantly for this study, fluorescence-based methods such as FLIM-FRET and BRET are difficult to implement in yeast microplate assays due to the high levels of cellular autofluorescence. NanoBiT bypasses this issue, providing a high signal-tonoise ratio.

Cons: Unlike FRET, NanoBiT requires the application of a substrate (furimazine). We did not include this disadvantage in the manuscript because it is not critical in a yeast study. Furimazine can be applied directly to the medium and readily permeates cells.

The authors state that the method is semi-quantitative - can they document this?

The semi-quantitative nature of the system is supported by its high dynamic range and the linear relationship between the luminescence signal and the amount of protein complex formed, as documented in Dixon et al. (2016). By using this system in a microplate setting, we were able to monitor relative increases or decreases in interaction levels over time across multiple metabolic conditions, providing a robust comparative analysis of metabolon dynamics.

All of the conclusions are based on the quality of this method. I know that it has been used by others, but at least some preliminary documentation to address these questions is required.

We acknowledge the reviewer’s concern regarding the reliance on the NanoBiT system. To ensure the reliability of our conclusions, we have included several lines of evidence to validate the method and demonstrate that the observed luminescence signals accurately reflect protein-protein interaction dynamics.

To confirm the NanoBiT results using an independent biochemical approach, we performed an in vivo pull-down assay following glucose addition (Figure 2 – figure supplement 1A). The results demonstrate a reduction in the physical association between MDH1 and CIT1. This biochemical validation directly supports the reduction in interaction observed with the NanoBiT system during the Crabtree effect.

We have provided protein abundance data for both MDH1 and CIT1 across the experimental conditions (Figure 2 – figure supplement 1&3; Figure 3 – figure supplement 1; Figure 4 – figure supplement 2). These results show only minor changes in protein levels, confirming that the fluctuations in the NanoBiT signal are independent of protein expression and represent genuine changes in metabolon assembly.

To ensure the findings are reproducible, we have included MDH1-CIT1 interaction results from repeated independent experiments (Figure 2 – figure supplement 1&3; Figure 3 – figure supplement 1; Figure 4 – figure supplement 1). The consistency of the results across these trials confirms the robustness of the system in monitoring the metabolic regulation of this complex.

We hope that these additional experimental validations, alongside the detailed technical description based on the established properties of the NanoBiT system (Dixon et al., 2016; Hall et al., 2012), provide the necessary documentation to satisfy the reviewer’s concerns regarding the quality and reliability of the method.

**Reviewer #2 (Public review):**

This study explores the dynamic association between malate dehydrogenase (MDH1) and citrate synthase (CIT1) in *Saccharomyces cerevisiae*, with the aim of linking this interaction to respiratory metabolism. Utilizing a NanoBiT split-luciferase system, the authors monitor protein-protein interactions in vivo under various metabolic conditions.

Major Concerns:

(1) NanoBiT Signal May Reflect Protein Abundance Rather Than Interaction Strength

In Figure 1C, the authors report increased MDH1-CIT1 interaction under respiratory (acetate) conditions and decreased interaction during fermentation (glucose), as indicated by NanoBiT luminescence. However, this signal appears to correlate strongly with the expression levels of MDH1 and CIT1, raising the possibility that the observed luminescence reflects protein abundance rather than specific interaction dynamics. To resolve this, NanoBiT signals should be normalized to the expression levels of both proteins to distinguish between abundance-driven and interaction-driven changes.

We agree that distinguishing between abundance-driven and interaction-driven changes is vital. To address this, we have included new data showing the relative protein levels of MDH1 and CIT1 across all experimental conditions. The protein levels were assessed using yeast lines expressing these proteins tagged with full-length NanoLUC luciferase (Figure 2 – figure supplement 1&3, Figure 3 - figure supplement 1, Figure 4 – figure supplement 2). Using the luminescence data of these relative protein levels, we have included plots showing normalized interaction index (Figure 2 – figure supplement 1G & 3D,H,L; Figure 3 - figure supplement 1D,H,L P; Figure 4 – figure supplement 1D,H,L). This index was calculated by dividing the NanoBiT interaction signal by the product of the relative abundances of both proteins:

$$\displaystyle \rm Normalized\, Interaction\, Index=\frac{NanoBit}{MDH1*\, CIT1}$$

In this formula, NanoBiT, MDH1, and CIT1 are the relative luminescence levels at each time point. This analysis clarified that the changes in the interaction signal significantly exceeded the fluctuations in protein levels, confirming that the dynamics are interactionspecific and not abundance-driven. To provide the most direct and transparent representation of the experimental measurements, we have chosen to keep the raw RLU data in the main figures and have moved the data related to protein abundance and normalization to figure supplements.

(2) Lack of Causal Evidence

The study presents a series of metabolic perturbation experiments (e.g., arsenite, AOA, antimycin A, malonate) and correlates changes in metabolite levels with NanoBiT signals. However, these data are correlative and do not establish a functional role for the MDH1CIT1 interaction in metabolic regulation. To demonstrate causality, the authors should implement approaches to specifically disrupt the MDH1-CIT1 interaction. One strategy could involve using a 15-residue peptide (Pept1) derived from the Pro354-Pro366 region of CIT1, previously shown to mediate the interaction, or introducing the cit1Δ3 (Arg362Glu) mutation, which perturbs binding. Metabolic flux analysis using ^13C-labeled glucose and mitochondrial respiration assays (e.g., Seahorse) could then assess functional consequences.

We agree with the reviewer that the current dataset correlates metabolon assembly with metabolic states rather than establishing a direct causal proof of its functional role in regulating pathway flux.

However, the primary objective of this manuscript was to establish the dynamic nature of the MDH1-CIT1 metabolon and to demonstrate the causal relationship between the changes in cellular conditions and metabolon dynamics through in vitro and in vivo assessments. Demonstrating that this canonical multienzyme complex undergoes reversible assembly and disassembly in vivo represents a major advance, as metabolon dynamics is a critical, yet previously unrevealed, factor involved in metabolic regulation. We aimed to define the specific environmental triggers that govern these dynamics, providing the necessary foundation for defining the functions of metabolons.

We completely agree that establishing causality using interaction-deficient mutants coupled with metabolic flux analysis is another critical experiment to establish the functions of the TCA cycle metabolon. We have, in fact, been conducting these precise metabolic flux analyses on CIT1 mutants with disrupted interaction with MDH1. Because the functional consequences of complex disruption involve wide-reaching metabolic rerouting that requires extensive data presentation and modeling, this work forms a separate, comprehensive follow-up study that is currently in preparation for submission in the near future.

To address this limitation in the current manuscript, we have carefully reviewed and revised the Abstract, Results, Discussion, and Conclusion sections (Lines 19-22; 205; 322-327; 341-342; 458-466). We have removed any language that may have inadvertently implied direct causality. We now explicitly state that our findings indicate the relationship between metabolon dynamics and respiratory conditions, and we have added a clear statement noting that the direct effects of this assembly on metabolic flux are the focus of our forthcoming studies.

(3) Absence of Protein Expression Controls Under Perturbation Conditions

In experiments involving acetate, arsenite, AOA, antimycin A, and malonate, the authors infer changes in MDH1-CIT1 association based solely on NanoBiT signals. However, no accompanying data are provided on MDH1 and CIT1 protein levels under these conditions. This omission weakens the conclusions, as altered expression rather than interaction strength could underlie the observed luminescence changes. Immunoblotting or quantitative proteomics should be used to confirm constant protein expression across conditions.

In response to your first concern, we have now performed protein expression assessments for all experiments, including the perturbation conditions, such as acetate, arsenite, AOA (Figure 3 – figure supplement 1), antimycin A, cyanide, and malonate (Figure 4 – figure supplement 2). The results demonstrate that the protein levels of MDH1 and CIT1 remain relatively stable throughout these treatments and do not correlate with the large changes observed in the interaction signals. This is also demonstrated by the normalized interaction index, which confirms that the shifts in luminescence are driven by the dynamic assembly and disassembly of the MDH1-CIT1 metabolon rather than changes in protein concentrations.

Conclusion:

Although the central question is compelling and the use of NanoBiT in live cells is a strength, the manuscript requires additional experimental rigor. Specifically, normalization of interaction signals, introduction of causative perturbations, and validation of protein expression are essential to substantiate the study's claims.

We sincerely thank the reviewer for recognizing the value of our central question and the strength of the live-cell NanoBiT system, as well as for your rigorous critique that has strengthened this manuscript. To address the concerns regarding experimental rigor, we have now provided extensive validation of MDH1 and CIT1 protein expression across all experimental conditions using yeast lines tagged with the full-length NanoLUC luciferase. These data demonstrate relatively stable protein expression, allowing us to calculate a normalized interaction index that substantiates that the observed luminescence shifts are driven by dynamic metabolon assembly rather than protein concentration. Regarding causative perturbations, we agree that introducing interaction-deficient mutants coupled with isotopic flux analysis is the critical next step to establish functional consequences. Because defining these pathway-wide rerouting events requires extensive modeling, this work will be reported in a follow-up study currently in preparation. Accordingly, we have carefully revised the manuscript to remove language implying direct causality, explicitly framing metabolon dynamics as an integral factor in metabolic regulation closely related to pathway activity and cellular metabolic states. We believe these new quantitative controls, normalizations, and textual clarifications thoroughly address the need for additional rigor and solidly substantiate our findings.

**Reviewer #3 (Public review):**

Summary:

Metabolons are multisubunit complexes that promote the physical association of sequential enzymes within a metabolic pathway. Such complexes are proposed to increase metabolic flux and efficiency by channeling reaction intermediates between enzymes. The TCA cycle enzymes malate dehydrogenase (MDH1) and citrate synthase (CIT1) have been linked to metabolon formation, yet the conditions under which these enzymes interact, and whether such interactions are dynamic in response to metabolic cues, remain unclear, particularly in the native cellular context. This study uses a nanoBIT protein-protein interaction assay to map the dynamic behavior of the MDH1-CIT1 interaction in response to multiple metabolic stimuli and challenges in yeast. Beyond mapping these interactions in real time, the authors also performed GC-MS metabolomics to map whole-cell metabolite alterations across experimental conditions. Finally, the authors use microscale thermophoresis to determine components that alter the MDH1-CIT1 interaction in vitro. Collectively, the authors synthesize their collected data into a model in which the MDH1CIT1 metabolon dissociates in conditions of low respiratory flux, and is stimulated during conditions of high respiratory flux. While their data largely support these models, some key exceptions are found that suggest this model is likely oversimplified and will require further work to understand the complexities associated with MDH1-CIT1 interaction dynamics. Nonetheless, the authors put forth an interesting and timely toolkit to begin to understand the interaction kinetics and dynamics of key metabolic enzymes that should serve as a platform to begin disentangling these important yet understudied aspects of metabolic regulation.

We thank the reviewer for this thoughtful and constructive summary of our work. We appreciate the recognition of the novelty and utility of our experimental approach and the integrated analysis of MDH1–CIT1 interaction dynamics.

We agree with the reviewer that, although our data largely support a model in which MDH1– CIT1 interaction correlates with respiratory activity, there are conditions that do not fully conform to this simplified framework. In the revised manuscript, we have addressed these apparent inconsistencies by providing detailed interpretations of the counterintuitive observations (e.g., ETC inhibition) and emphasizing that the MDH1–CIT1 interaction is modulated by changes in the mitochondrial matrix microenvironment associated with respiratory activity.

Furthermore, we have revised the Discussion to highlight that the regulation of the MDH1– CIT1 interaction is likely multifactorial, involving the combined effects of pH, metabolites, and other unknown factors, which together enable fine-tuning of metabolic flux in fluctuating environments. This expanded perspective is now more clarified.

We agree that identifying the precise molecular determinants of MDH1–CIT1 interaction dynamics will require additional mechanistic studies, such as systematic analyses using yeast mutants. While these experiments are an important next step, they are beyond the scope of the present study. We anticipate that the toolkit and framework established here will facilitate such future investigations.

Strengths:

(1) The authors address an important question: how do metabolon-associated proteinprotein interactions change across altered metabolic conditions?

(2) The development and validation of the MDH1-CIT1 nanoBIT assay provides an important tool to allow the quantification of this protein-protein interaction in vivo. Importantly, the authors demonstrate that the assay allows kinetic and real time assessment of these protein interactions, which reveal interesting and dynamic behavior across conditions.

(3) The use of classic biochemical techniques to confirm that pH and various metabolites can alter the MDH1-CIT1 interaction in vitro is rigorous and supports the model put forth by the authors.

We thank the reviewer for these positive and encouraging comments. We are pleased that the importance of the research question, the development of the MDH1–CIT1 NanoBiT assay, and the integration of in vivo and in vitro approaches were recognized. We especially appreciate the acknowledgment of the assay’s ability to capture dynamic and kinetic changes in protein–protein interactions, as well as the support provided by the biochemical analyses. We hope that the experimental framework established in this study will serve as a useful platform for further investigations into metabolon dynamics and metabolic regulation.

Weaknesses:

(1) Some of the data collected seem to be merely reported rather than synthesized and interpreted for the reader.

We agree that explicitly synthesizing these findings is essential for clarity. To improve this, we have revised the Results section to include concise summary statements at the conclusion of each major experimental paragraph (Lines 190-191, 201, 218-219, 229-231, 241-242, 272-274, 282-283; 291-293). These additions interpret the data in relation to our main hypothesis. The discussion section was thoroughly revised to more precisely explain the logic supporting the model (Lines 381-393; 433-443, 458-466). Additionally, to bring together the entire dataset, we introduced a new summary schematic (Figure 6A). This figure visually and conceptually integrates our diverse findings, covering metabolic treatments, pH fluctuations, and complex metabolite profiles, showing how these signals work together to control multienzyme complex assembly.

This is particularly true for data that seem to reflect more complex trends, such as the GCMS experiments that map metabolites across multiple experiments, or treatments that show somewhat counterintuitive results, such as the antimycin A treatment, which promotes rather than disrupts the MDH1-CIT1 interaction.

We agree that our complex datasets, including the metabolomics and the seemingly counterintuitive Antimycin A results, required deeper synthesis. To clarify the broader metabolic trends, we have added Figure 6A to visually map which factors, specifically pH, malate, fumarate, and aspartate, most consistently align with complex assembly. We revised the Discussion (Lines 390-393, 439-443) to explicitly conclude that no single variable predominantly governs the interaction, but it is coordinately regulated by multiple microenvironmental cues.

Regarding the Antimycin A (and other ETC inhibitors) discrepancy, where the interaction is enhanced despite suppressed respiration, we have expanded our interpretation (Lines 346–358) to explain this as a transient response that is not directly reflected by steadystate respiratory activity. Specifically, we propose that acute perturbations of the mitochondrial matrix microenvironment, particularly changes in pH, temporarily promote MDH1–CIT1 interaction. Thus, under these conditions, transient microenvironmental changes can dominate over steady-state respiratory output in regulating metabolon assembly.

The discussion paragraph about the imperfect relationship between pH and interaction has been revised to highlight our conclusion that mitochondrial matrix pH can be a contributing factor rather than the primary regulator (Lines 386-393).

(2) Some of the assertions put forth in the manuscript are not substantiated by the data presented, and the authors are at times overly reliant on previous findings from the literature to support their claims. This is particularly notable for claims about "TCA cycle flux"; the authors do not perform flux analysis anywhere in their study and should be cautious when insinuating correlations between their observations and "flux".

We appreciate the reviewer’s careful evaluation of our terminology and fully agree that claims regarding "flux" should be reserved for studies that employ direct isotopic flux measurements. In response to this constructive feedback, we have thoroughly reviewed the manuscript to ensure that our assertions are substantiated by the presented experimental data. We have carefully evaluated the use of the term "flux" throughout the Abstract, Introduction, and Discussion, replacing it with more accurate phrases such as "pathway activity," "respiratory activity," or "mitochondrial respiration" depending on the specific context (Lines 11; 20-21; 50; 111-112; 322-327; 329; 345; 349-350; 442-443; 458466).

We also removed a paragraph discussing the potential role of the MDH1-CIT1 metabolon in the malate-aspartate shuttle (Line 361). We realized the paragraph is highly speculative, and our data do not directly support the hypothesis. The influence of the MDH1-CIT1 on the malate-aspartate shuttle is a major finding of the upcoming manuscript reporting its effects in metabolic network flux. We apologize for mixing up the results of two separate studies.

Furthermore, we have revised our conclusions to avoid over-reliance on prior literature in making causal claims. We now explicitly frame the dynamic assembly of the MDH1-CIT1 metabolon as an integral factor in metabolic regulation, closely related to cellular metabolic states, rather than stating that it controls pathway flux (Lines 454-462). We believe these textual revisions accurately align our claims with our current observations and remove any unsubstantiated assertions.

(3) The manuscript presentation could be improved. For figures, at times, the axes do not have intuitive labels (example, Figure 1A), data points and details about the number of samples analyzed are missing (bar graphs and box plots), and molecular weight markers are not reported on western blots. The authors refer to the figures out of order in the text, which makes the manuscript challenging to navigate as a reader.

We thank the reviewer for these helpful suggestions to improve the clarity and presentation of the manuscript. We have made several revisions accordingly.

First, axis labels have been revised throughout the figures to improve clarity and make them more intuitive. Second, we have added the number of biological replicates to the figure captions and updated bar graphs and box plots to display individual data points. Third, to improve the transparency of the immunoblot data, we have included molecular weight marker position in Figure 1C and corresponding full gel images in a new Figure 1 – figure supplement 2. Other immunoblot images have been moved to Figure 2 – figure supplement 1 since they lack molecular marker images.

In addition, we have reorganized the figure panel labeling and corresponding text to improve the flow of the Results section. Specifically, figure subpanels are now arranged according to the measured parameters rather than treatment conditions, and the relevant sections describing TCA cycle manipulation and ETC inhibition have been revised to follow this updated figure order (Lines 208–231; 251–274). These changes improve the readability and logical progression of the manuscript.

**Recommendations for the authors:**

**Reviewer #1 (Recommendations for the authors):**

The grammar in the abstract in the sentence which states called metabolon. This needs to be fixed.

We thank the reviewer for pointing this out. We have revised the sentence in the Abstract to improve clarity. The revised sentence reads: “The tricarboxylic acid (TCA) cycle enzymes malate dehydrogenase (MDH1) and citrate synthase (CIT1) form a multienzyme complex, referred to as a metabolon, that channels intermediate oxaloacetate between their reaction centers.” (Lines 7-9)

**Reviewer #3 (Recommendations for the authors):**

Major points:

(1) Much of the data reported in this manuscript reads as a summary of what was found, rather than distilling what the trends in the data mean or how they support the proposed model.

We thank the reviewer for this comment. This concern overlaps with your previous point (Weakness 1), which we have addressed through revisions to improve synthesis and clarity. Specifically, we have added concise summary statements at the end of each major experimental section (Lines 190-191, 201, 218-219, 229-231, 241-242, 272-274, 282-283; 291-293), and we have included a new summary schematic (Figure 6A) that integrates the findings to illustrate how metabolic conditions and mitochondrial microenvironments relat to MDH1–CIT1 interaction. Together, these revisions improve the interpretation and clarify how the results support our model.

For instance, in Figure 3, the authors use one metabolic treatment to activate the TCA cycle and two to inhibit the TCA cycle. In Figure 3M, GC-MS data are reported for select metabolites across these three conditions, as well as a control condition. However, these metabolites don't follow clean "trends" according to the predictions; as one example, malate is down in the TCA active (acetate) and one TCA inhibited condition (arsenite), whereas it is elevated in the second TCA inhibited (aminooxyacetate) condition. As an additional example, glutamate is down in the arsenite (inhibited) condition, slightly down in the acetate (activated) condition, but is unchanged in the AOA (inhibited) condition. Similar variability is seen in Figure 4M. What do these discrepancies mean? How do they support the model? As written, these data bring forth more questions than they answer.

We appreciate the reviewer’s careful analysis of the metabolomics data in Figures 2E, 3M, and 4M. The reviewer notes that the levels of certain metabolites show complex patterns that do not simply reflect overall TCA cycle activity. We have acknowledged that our metabolomics dataset is a valuable resource for the research community and have added a brief paragraph to emphasize the complex metabolic phenotypes resulting from chemical treatments (Lines 422-431).

As mentioned in the paragraph, this complexity is biologically expected. It is likely from the distinct primary targets of each inhibitor, such as arsenite affecting redox-sensitive enzymes and AOA disrupting the malate-aspartate shuttle, as well as off-target effects and the adaptive reorganization of intersecting metabolic networks to bypass local blockades. Rather than viewing these diverse metabolic phenotypes as discrepancies, we leveraged them to uncouple general respiratory suppression from specific metabolite pools, allowing us to independently assess their relationship with metabolon assembly.

Furthermore, we note that our GC-MS analysis measures whole-cell metabolite levels, which represent the sum of multiple subcellular compartments and may not precisely reflect localized concentrations within the mitochondrial matrix that is directly affected by the TCA cycle. The description of this limitation of whole-cell metabolomics has been revised in Lines 417-420.

(2) Why do the authors propose that antimycin A increases the interaction between MDH1 and CIT1 despite decreasing respiratory activity? Given the generalities proposed in Figure 6, this is important to address.

We thank the reviewer for this comment. This point overlaps with Weakness 1, where we have addressed the apparent discrepancy associated with antimycin A (and other ETC inhibitors). Briefly, we have expanded our interpretation (Lines 349–360) to explain this effect as a transient response that is not directly aligned with steady-state respiratory activity. We propose that acute perturbations of the mitochondrial matrix microenvironment, particularly changes in pH, temporarily promote MDH1–CIT1 interaction. In addition, we have revised the Discussion (Lines 386–404) to clarify that mitochondrial matrix pH acts as a contributing factor rather than the primary regulator of the interaction. Together, these revisions reconcile the ETC inhibition by antimycin A with the overall model presented in Figure 6.

(3) The authors use acetate to "activate" the TCA cycle; do other non-fermentable carbon sources also promote the MDH1-CIT1 interaction?

We thank the reviewer for this insightful question. We have tested additional nonfermentable carbon sources and found that they did not significantly affect MDH1–CIT1 interaction (Figure 3—figure supplement 1). We note that raffinose present in the medium likely provides a baseline carbon source supporting oxidative metabolism, which may limit the observable effects of these treatments (Lines 149-150).

In addition, we performed a new experiment using ethanol. While ethanol treatment enhanced the MDH1–CIT1 interaction signal, it also increased the abundance of MDH1 and CIT1, resulting in a reduced interaction index. Because ethanol induces protein accumulation under our experimental conditions, this result is not straightforward to interpret. We have included this observation and its interpretation in the revised manuscript (Lines 208–211).

(4) The authors show that the MDH1-CIT1 interaction is sensitive to pH. Is the MDH1-CIT1 interaction affected by uncouplers in vivo?

We thank the reviewer for suggesting a meaningful experiment. We performed a new experiment examining the effect of the uncoupler CCCP on MDH1–CIT1 interaction in vivo (Figure 4—figure supplement 4). We found that CCCP treatment increased the interaction signal, consistent with the idea that acidification of the mitochondrial matrix promotes MDH1–CIT1 association.

However, we observe that CCCP treatment also decreased the luciferase signals from MDH1 and CIT1 fused to full-length NanoLUC in an abnormal way, making it harder to interpret the interaction index. Therefore, although these results support a possible role for pH in regulating the interaction, they should be viewed with caution and included as a figure supplement. This experiment and its interpretation have been added to the revised manuscript (Lines 276–283).

(5) NADH is a potent suppressor of many enzymes within the TCA cycle, including MDH1 and CIT1. Can the authors modulate mitochondrial NADH through genetic manipulation of Ndi1, or through overexpression of mito-Lb-NOX (PMID: 27124460)?

We thank the reviewer for this insightful suggestion. We agree that the mitochondrial NADH is a potential regulator of the MDH1-CIT1 interaction as it is a potent suppressor of many TCA cycle enzymes, and indeed, we have previously shown that NADH inhibit the MDH-CS interaction in vitro (Omini et al 2021 PMID: 34548590). For this reason, we investigated the mitochondrial matrix redox state that is related to the NADH levels in the current study. The reviewer’s proposed strategy of using targeted genetic tools like mito-Lb-NOX or Ndi1 manipulation to specifically influence the NADH level is an elegant approach to isolate this variable. However, implementing this system requires generating, optimizing, and validating new yeast strains that harbor the targeted NADH-modulating constructs alongside NanoBiT and full-length NanoLUC sensor systems. Because this extensive strain engineering and subsequent live-cell validation fall outside a feasible timeframe for the current manuscript revision, we must respectfully defer these experiments. We view the precise manipulation of the mitochondrial redox state via tools like mito-Lb-NOX as a complementary approach for our future work to systematically pinpoint the individual regulatory factors. We have expanded our Discussion (Lines 417-420; 462-465) to highlight the targeted genetic manipulation of the possible regulatory factors including the NADH pool, as a critical future direction for dissecting these dynamics.

(6) The authors should correct their figures:

(a) Axes should be easy to interpret on graphs.

(b) Individual datapoints should be shown on bar graphs and box plots. Minimally, the number of samples evaluated should be reported.

(c) Molecular weight markers should be reported on blots.

We thank the reviewer for these helpful suggestions. Points (a) and (b) overlap with Weakness 3, which we have addressed through revisions to improve figure clarity and data presentation. Specifically, axis labels have been revised to be more intuitive, the number of samples is now reported in the figure captions, and bar and box plots have been updated to include individual data points. For time-course data, we retained point-line plots, as alternative formats (e.g., bar or box plots) would reduce clarity due to the density of time points.

For point (c), we have added molecular weight markers to the immunoblot data where available (Figure 1C). In the time-course experiment in the original Figure 2, molecular weight markers were absent from the gel images. Although we are confident in the identity of the detected signals, we have moved these data to a figure supplement (Figure 2—figure supplement 1C) to reflect this limitation. Similarly, the corresponding Co-IP data are now presented as a figure supplement (Figure 2—figure supplement 1A).

Minor points:

(1) In the last paragraph before the results, the authors refer to "the fluorescent biosensors", but start the paragraph discussing the nanoBIT PPI. After reading the manuscript, these seem to be distinct experimental setups, but that was not evident in the first read through of the paper.

We thank the reviewer for pointing out this source of confusion. We apologize for the lack of clarity in distinguishing between the experimental approaches. In this study, the NanoBiT system was used to measure MDH1–CIT1 interaction, whereas fluorescent biosensors were used to assess mitochondrial matrix pH, redox state, and ATP levels. We have revised the paragraph to more clearly distinguish these methodologies and their respective roles in the study (Lines 105–112).

(2) As mentioned above, referring to multiple figures out of order within the manuscript is very jarring for the reader. The authors should consider reworking the narrative or figures to be presented in order.

We thank the reviewer for this comment. This concern overlaps with the previous comment regarding figure organization, which we have addressed by revising both the figure labeling and the corresponding text. Specifically, figure subpanels have been reorganized to follow the measured parameters rather than treatment conditions, and the Results sections describing TCA cycle manipulation and ETC inhibition have been revised to follow the updated figure order (Lines 208–231; 251–274). These changes improve the logical flow and readability of the manuscript.
